# Examining the Intention of Authorization via Apps: Personality Traits and Expanded Privacy Calculus Perspectives

**DOI:** 10.3390/bs12070218

**Published:** 2022-06-29

**Authors:** Jie Tang, Bin Zhang, Shuochen Xiao

**Affiliations:** 1 School of Economics and Management, Beijing University of Posts and Telecommunications, Beijing 100876, China; jietang@bupt.edu.cn (J.T.); binzhang@bupt.edu.cn (B.Z.); 2 School of Economics and Management, Hebei University of Architecture, Zhangjiakou 075000, China

**Keywords:** privacy calculus, trust, personality traits, prior negative experience, App users

## Abstract

By integrating the extended privacy calculus theory with the Big Five personality theory, this research proposes and validates a conceptual model in the context of mobile application (App) information authorization. It investigates the implications of each component of privacy costs, privacy advantages, and trust on users’ willingness to authorize their information, and explores how the five personality traits affect App users’ perceived benefits, privacy concern, and trust. Simultaneously, the links between prior negative experience and privacy concern as well as the final authorizing willingness were uncovered. We employed a questionnaire to collect 455 users’ data, and the partial least squares structural equation model (PLS-SEM) was used to test the hypotheses. The findings demonstrate that App users’ perceived benefits and trust have a positive impact on their privacy authorization intention, whereas privacy concerns negatively affect their disclosure willingness. Just as Extraversion and Agreeableness would make someone pay a heightened attention to the benefits, agreeable, neurotic, and conscientious users are more easily stimulated by privacy concern. Respectively, Agreeableness and Neuroticism affect users’ trust positively and negatively. Additionally, prior negative experience will trigger an individual’s privacy concern, which in turn hinders their willingness to authorize his/her information. All of the aforementioned can serve as a guide for App providers as they optimize the features of their products and services, implement the necessary privacy protections to alleviate users’ privacy concern, and boost users’ trust belief. More importantly, these results effectively demonstrate the significance of personal traits in the formation of users’ privacy perceptions.

## 1. Introduction

Mobile applications (Apps) serve as a vital link between users and the mobile network, transporting a significant amount of data. With data becoming a highly dependent production factor for corporate operations, the maximum collection of user information has also become a business consensus, and issues such as mandatory claims of rights, excessive collection, and the use of user information by enterprises through apps are becoming increasingly prominent. Because of widespread privacy concern, an increasing number of users are refusing to give applications personal information or claiming privacy rights by faking personal data. According to the KPMG 2021 report, Corporate Data Responsibility: Bridging the Consumer Trust Gap, 86 percent of respondents believe that data privacy issues cause a decline in corporate trust, and 68 percent believe that the level of data collection by companies is a concern, indicating that closing the trust gap will be challenging [1]. Users’ concern about disordered personal privacy standards are triggered by privacy issues, which deteriorates the Apps’ ecology and obstructs the industry’s healthy development. As a result, App providers and the entire industry must investigate issues such as understanding users’ privacy views, minimizing users’ privacy concern, and increasing users’ willingness to authorize their personal information.

Individual information behavior and the factors that influence it have been the subject of information systems (IS) research. Privacy calculus theory is a common theory in information security research that examines various user decision-making factors in terms of privacy trade-offs [2,3,4]. In comparison to typical Internet applications, mobile Apps communicate a considerable amount of user data in real time, which increases the probability of privacy issues such as excessive information gathering and exposure [5]. Furthermore, most of the existing studies on privacy calculus focus on a single scenario, resulting in incredibly specific perceived cost and benefit aspects, as well as a lack of universality in privacy calculus models. Based on the aforementioned limitations, we attempt to assess individuals’ perceived benefits and costs in the privacy authorization process from a broader perspective, and we follow the research ideas of Dinev and Hart [6] and Thompson and Brindley [7] by adding a trust dimension to the model to investigate the joint impact of costs, benefits, and trust on users’ privacy decisions. Meanwhile, considering App users are not a homogeneous group, this paper attempts to use the Big Five personality traits and past negative experiences as the antecedent variables of the model, to investigate the driving or constraining effects of various personality traits and prior experiences on users’ privacy perceptions.

The objective of this research is to provide evidence-based answers to the following questions: How do personality traits influence App users’ privacy-related constructs when information authorization is required? What effect does the extended privacy calculus model have on the willingness of App users to authorize personal information?

Three theoretical or practical aims are expected to be met as a result of this research. First, an attempt is made to develop a more thorough privacy decision model that illustrates and explains the many factors that affect the authorizing intention of users’ personal information. Second, this study sets out to gain a better understanding of App users’ privacy decision intentions at the individual level, as well as to demonstrate how the Big Five model can be combined with privacy calculus theory and trust theory. Third, on a practical level, this study will provide App providers with a greater grasp of users’ privacy awareness and perceptions, which will assist enterprises in improving their privacy practices and industry regulatory authorities in formulating appropriate regulatory policies to promote the healthy and orderly development of the mobile App industry.

## 2. Theoretical Background

### 2.1. Personal Data, Personal Information, and Privacy

In both theory and practice, the terms “personal data,” “personal information,” and “privacy” are used, conveyed, and defined in an ambiguous way [8]. The General Data Protection Regulation of the European Union (GDPR) defines “personal data” as “any information related to an identified or identifiable natural person” and broadens the definition to include IP addresses and cookie data [9]. The Personal Information Protection Law of China uses the term “personal information,” although it has a connotation that is nearly identical to that of “personal data” as it is defined by the GDPR. In contrast, data or information privacy is weighted more heavily under US law [10,11]. The phrases “privacy” and “personal information” are frequently used interchangeably in the context of privacy decision-making studies [12,13,14], which concentrate on users’ attitudes and willingness to disclose or share their personal information rather than specific notions. Hence, this study defers to the idea that, broadly speaking, personal information includes private information and that privacy is essentially an informational concept [15].

In academic research, the meaning of privacy varies depending on the research environment; hence, there is no definitive definition. According to Hann et al. [16], an individual’s awareness and control over the gathering and use of his/her personal data by others constitute privacy in the online environment. Additionally, the control over information about oneself is considered as personal information privacy [17]. A key component of privacy is decisional autonomy, which Westin [18] defined as the capacity to decide when, where, and to what extent people expose their views or activities. Wheeless and Grotz [19] described information disclosure, commonly referred to as “self-disclosure,” as any information a person shares with others about themselves. The authorization of personal information discussed in this paper refers to the actions taken by mobile App users who voluntarily provide personal information to service providers in order to fulfill their functional requirements and permit those providers to collect, store, and use the information in the ways specified by law.

### 2.2. Privacy Calculus

From an economic standpoint, privacy calculus is one of the most effective frameworks for contemporary privacy research, demonstrating the combined role of opposing elements—costs and benefits—on privacy perceptions and actions [2,3,4]. This theory views privacy as a commodity with transactional features, where personal information is provided in exchange for something valuable, like improved services [20]. Specifically, users’ disclosure of personal information will be accompanied by a set of potential opportunistic risks, such as illegal collection, illegal transaction, and accidental disclosure of information, all of which are regarded as the costs of their self-disclosure. Meanwhile, individuals will gain functional benefits [21,22], financial benefits [23], or social benefits [24] from the circulating data used by information receivers, hence the benefits of information disclosure emerging. The above analysis proves that someone will employ “privacy” to carry out certain rights and interest exchanges in order to enhance self-utility [25]. Without considering cognitive bias and information asymmetry, when the total perceived benefits of disclosure exceed the total costs, there is often a willingness to sacrifice some privacy for different types of benefits, which is also a finding that provides a reasonable explanation for the ambiguous “privacy paradox” phenomenon.

As it is difficult to assess the costs or benefits directly, users will weigh the pros and cons of privacy disclosure based on their own subjective judgment. According to Li et al. [26], during online transactions, interaction benefits primarily consist of consumers’ perceived usefulness and monetary rewards, whereas their perceived costs primarily consist of privacy protection beliefs and privacy risk beliefs. Wang et al. [27] divided the perceived benefits of disclosure for social media users into monetary rewards and social rewards, whereas the cost aspect is quantified through privacy concern. When accepting permission requests, Wottrich et al. [5] considered intrusiveness and privacy concern as the costs associated with information transactions, and the perceived value of the app as the overall benefits to the user, providing an in-depth study of the privacy trade-offs made by users during the download or use of mobile Apps. In addition, Appendix A contains a list of common cases for privacy calculus research.

In summary, the reference factors of the perceived cost are largely people’s perception of privacy risk or privacy concern, both of which are risk beliefs. Perceived privacy risk, on the other hand, is the user’s overall assessment of the online environment’s risk profile, whereas perceived privacy concern is the user’s overall estimate of the potential negative effects of sharing personal information online [6]. According to Wang et al. [28], users’ privacy concern is determined by a combination of their perceived privacy risks and their ability to cope with prospective dangers. As a result, we suggest that privacy concern, rather than perceived privacy risk, provides a more comprehensive reaction to the potential losses that users confront during the privacy decision process. In line with Pentina et al. [29], the sum of App users’ perceived benefits in the context of privacy authorization were treated as a second-order variable. Additionally, trust plays a significant role in social exchange theory, which is where privacy calculus framework originated [30]. Dinev and Hart [6], Premazzi et al. [31], and Krasnova. et al. [32] addressed trust as an epistemic component of the privacy calculus theory. Simultaneously, the facilitative nature of trust on users’ privacy disclosure decisions was pointed out. In this paper, we draw on previous research and integrate the trust belief with privacy calculus theory to investigate the impact of the three aspects (costs, benefits, and trust) on App users’ willingness to authorize personal information.

### 2.3. Personality Traits

Personality traits are persistent psychological characteristics of individuals that can impact their thoughts, feelings, and actions in the outside world [33,34]. Researchers have long disagreed over how to classify and describe personality traits, such as Cattell’s 16 basic personality traits [35] and Eysenck’s three personality dimensions [36]. Until the 1980s, the Big Five personality model evolved into an accepted theory of personality research among psychologists [37,38,39]. By integrating a number of overly fragmented personality dimensions, the Big Five model summarized five distinctive and unrelated personality traits of Neuroticism, Extroversion, Openness to experience (Openness), Agreeableness, and Conscientiousness. Meanwhile, a standardized scale NEO-Personality-Inventory (NEO-PI) was developed [39]. Agreeableness and Conscientiousness reflect interpersonal dimensions. Extraversion represents temperamental component. The emotional dimension is symbolized by Neuroticism, and Openness to experience is significantly associated with cognition.

In the fields of individual motivation and decision-making behavior, multiple studies have demonstrated that the five personality traits play a significant role in the formulation of perception and behavioral intention [40,41,42]. Smith et al. [43], in a related study in the field of information systems, were the first to show that users are not a homogeneous population with widely divergent privacy perceptions and attitudes. Some people are unconcerned with privacy, while others are sensitive about it. Following this, personality qualities have been incorporated into numerous privacy models; for instance, Bansal et al. [44] proposed the TRA-privacy framework and confirmed that different personality traits have distinct effects on users’ trust and privacy concern. Pentina et al. [29] compared the influence of the Big Five personality traits on the perceived benefits and privacy concern of mobile App users in China and the United States. The Big Five personality theory was combined with the TAM model to explore the influence of personality traits on users’ acceptance of mobile commerce by Zhou and Lu [45]. The framework of current studies on personality traits and privacy-perception factors has been collated and is given in Table 1 below. These findings show that people’s privacy concern, perceived benefits, and trust beliefs about new technology can all be explained to some extent by personality features.

## 3. Research Model and Hypotheses

### 3.1. Effects of Personality Traits on Privacy Calculus and Trust

Goldberg [54] defined extraversion as a personality attribute that shows humanism, including key words such as talkative, optimistic, daring, and confident. People with this personality feature are more likely to have utilitarian intentions, be more eager to accept new things [55], participate in social activities [56], and be more conscious of the hedonic and utilitarian value of new objects [57]. Pentina et al. [29], for example, proposed that mobile App users’ extraversion boosted their perception of an App’s benefits. According to the conclusion of Mouakket and Sun [49], extraversion also had a positive association with the utilitarian value of SNSs. There is a negative association between extraversion and privacy concern [48,58,59] or no significant relationship [40,50,60]. Furthermore, Walczuch and Lundgren [61] discovered a link between extraversion and trust in online services. Zhou and Lu [45] revealed that extraverted mobile commerce customers are more inclined to trust service providers during interactions. Based on the above analysis, the following hypotheses are proposed:

**H1a.**  *Extraversion positively affects App users’ perceived benefits;*

**H1b.**  *Extraversion negatively affects App users’ privacy concerns;*

**H1c.**  *Extraversion positively affects App users’ trust.*

Politeness, friendliness, tolerance, and helpfulness are key words for agreeableness [62]. People with this trait tend to have a more moderate mindset to reduce conflicts with others and the outside world. When faced with new technologies or products, their tolerant and receptive nature allows them to focus on the positive aspects [29,34,63]. Mouakket and Sun [49] uncovered that highly-agreeable users are more likely to consider SNS as useful and enjoyable. Furthermore, people with the agreeableness trait are better at building trust and friendly relationships with others [41,44,45,64], and the recognition of establishing a just and equitable social order make them deeply concerned about privacy invasions [48,65]. Conversely, it has been discovered that users or consumers with agreeableness are less sensitive to privacy threats, and this personality trait will appropriately mitigate privacy concern, according to Junglas et al. [40]. Here, we argue that affable users have a positive and tolerant attitude toward information requests from App providers and are more trusting of the data protection environment and the companies they work with, but they are also concerned about privacy issues and will be extra vigilant to potential negative consequences. It is, thus, hypothesized:

**H2a:**  *Agreeableness positively affects App users’ perceived benefits;*

**H2b:**  *Agreeableness positively affects App users’ privacy concern;*

**H2c:**  *Agreeableness positively affects App users’ trust.*

Neuroticism or emotional instability is characterized by anxiety, suggestibility, and impulsivity [66]. Individuals with this personality trait have frequent mood swings, a poor ability to cope with external stressors, and a tendency to focus on the negative repercussions and potential losses of events [67], all of which make building trust with the outside world more challenging [68]. Most studies have also shown that neurotics frequently suffer from cyber anxiety, which will not only cause them to be overly concerned about privacy during new technology adoption or information disclosure [44,50], but will also affect their positive views of new technologies or services, as Zhou and Lu [45], Uffen et al. [69], and Agyei et al. [47] confirmed. In the study of personality traits and trust, Pour and Taheri [51] discovered that neuroticism was negatively connected with students’ perceptions of trust and knowledge sharing behavior with SNS. In this research, we claim that a high level of anxiety and terror would prevent users from creating confidence with App providers, and their privacy concern when permitting personal information would skyrocket, while perceived benefits from information concessions would plummet, assuming that:

**H3a:**  *Neuroticism negatively affects App users’ perceived benefits;*

**H3b:**  *Neuroticism positively affects App users’ privacy concern;*

**H3c:**  *Neuroticism negatively affects App users’ trust.*

In contrast to immaturity, impatience, and impulsiveness, conscientious people are reliable and cautious, with a strong feeling of personal accomplishment [62]. People with this personality trait have a strong sense of privacy protection and risk avoidance [70]. They are inclined to evaluate the possible hazards of disclosing private information while making privacy decisions and are very concerned about privacy issues [40,58]. Simultaneously, conscientious users will have a better understanding of the advantages, whether the usefulness perception or the enjoyment perception, of disclosing information [47,71]. Furthermore, several studies have revealed that conscientiousness influences the trust level of individuals, whereas the conclusions are not unique. It was, for example, found by Bawack et al. [53] that dutiful users are more likely to trust a smart speaker voice-guided service that provides a positive experience. Nevertheless, Pour and Taheri [51] found that conscientiousness leads to a lower disposition to trust, while Zhou and Lu [45] and Bansal et al. [44] proved that there is no significant relationship between conscientiousness and trust. In the context of App privacy authorization, we hypothesize that people with traits of conscientiousness will be especially cautious in their decisions, which implies that they will weigh the various benefits of authorizing personal information, be more concerned about personal privacy information, and have a higher trust threshold with the App provider. These are the hypotheses:

**H4a:**  *Conscientiousness positively affects App users’ perceived benefits;*

**H4b:**  *Conscientiousness positively affects App users’ privacy concern;*

**H4c:**  *Conscientiousness negatively affects App users’ trust.*

The proclivity to be creative, inventive, and adventurous has been termed as open to experience, sometimes referred to as intellect [72]. Individuals with this trait are intrigued by the unusual and reject regularity to some extent [66]. Numerous studies have shown that they are eager to disclose personal information in exchange for additional functions or services that they require [58,73,74]. From another point of view, they are also more rational and optimistic about the privacy threats associated with new technologies [40,75]. In terms of their personal trust, Zhou and Lu [45] noted that users with open personalities are more inclined to trust mobile service providers. Dinero and Chua [76] also demonstrated that openness leads to a higher level of trust during location-based information disclosure. According to the aforementioned reasoning, highly open users will have a more positive opinion of the benefits of the App they are about to use but will be less concerned about privacy issues. What is more, they will be more confident in authorizing their personal information. The hypotheses are:

**H5a:**  *Openness positively affects App users’ perceived benefits;*

**H5b:**  *Openness negatively affects App users’ privacy concern;*

**H5c:**  *Openness positively affects App users’ trust.*

### 3.2. Effects of Prior Negative Experience on Privacy Concern and Intention

Since an individual’s future prognosis is based on a subjective experience, his/her past experiences will affect their attitudes and perceptions, as well as subsequent behavioral decisions [77]. In this research, we examine the impact of the prior negative experiences, or what may be referred to as “privacy invasion events,” of App users on their privacy concern, such as the over-collection of personal data, data leaking, and criminal misuse. Prior negative experiences reduce personal controllability of outcomes and perceived availability [78]. As a result, users who have had negative experiences with privacy are more vigilant about their personal information, which would, in turn, trigger their privacy concern and lead to a reduction in their willingness to disclose personal information. Li et al. [79] revealed that privacy invasion experience is a significant factor in the establishment of privacy concern among SNS users, whereas Ampong et al. [80] showed that negative privacy experiences reduce social network users’ intention to disclose information. According to Metzger [81], consumers with E-commerce experience were more inclined to offer fake information to limit personal privacy disclosures. We propose the following hypotheses based on previous theoretical and empirical evidence:

**H6a:**  *Prior negative experience positively affect App users’ privacy concern;*

**H6b:**  *Prior negative experience negatively affect App users’ authorizing intention.*

### 3.3. Effects of Privacy Calculus on Intention to Authorize Personal Information

Individuals’ subjective expectations about the beneficial impacts of disclosing personal information, known as perceived benefits, are the extrinsic determinants of their privacy decisions. Individuals’ perceived benefits can take several different forms, including personalization [23], usability [26], entertainment [82], convenience [83], financial gain [23], and relationship management [84], all of which have a more marked positive impact on users’ personal information disclosure or new technology adoption. In this paper, we employ Pentina et al.’s [29] research dimension on perceived benefits to argue that by authorizing their personal information, mobile App users can obtain three needs: information source, leisure, and social interaction. Information sources include users’ access to information services that meet their needs through Apps, such as getting location information, retrieval information, product list information, and dynamic consultation information. Leisure focuses on the emotional experience of users, and includes games, music, short videos, and other Apps that can provide users with satisfaction and pleasure. The term “social interaction” refers to communication and exchanges with individuals and the outside world, and it primarily refers to Apps having social communication features (such as WeChat, Weibo, Tik Tok, etc.). In a study of App adoption and personal information disclosure, Wang et al. [85] discovered that App users’ perceived benefits positively affect their willingness to disclose information. Users will make privacy trade-offs when adopting or using mobile Apps, according to Wottrich et al. [5], and the perceived value of these Apps is the most important element influencing users’ authorization decisions. Furthermore, Wakefield [82] and Susanto et al. [86] both found that individuals’ perceived benefits are positively connected with trust in a given context.

**H7a:**  *Perceived benefits positively affect App users’ trust;*

**H7b:**  *Perceived benefits positively affect App users’ authorizing intention.*

The main hurdle to users’ information disclosure and the adoption of new technologies has been their privacy concern, which usually relate to individuals’ subjective expectations of a possible loss of privacy [17]. Some researchers viewed privacy concern as a multidimensional concept, such as Smith et al.’s CFIP model [43], Malhotra et al.’s IUIPC model [17], and Xu et al.’s MUIPC model [87], which all looked at the constructs of privacy concern from multiple sub-dimensions. Privacy concern is incorporated into each model as a unidimensional factor in the classic privacy research literature, such as by Dinev and Hart [6] and Xu et al. [72], with the goal of exploring the interaction between privacy concern and other factors. In this article, we apply Xu et al.’s [88] concept of privacy concern and research dimensions, which defines privacy concern as the potential loss of privacy caused by users’ authorization of personal information to App providers. Growing privacy concern may cause users to refuse to permit personal information disclosure, as Junglas et al. [40], Bansal et al. [44] and Yeh et al. [46] have confirmed. Furthermore, numerous studies show that users’ privacy concern is strongly linked to corporate distrust [13,17,89]. We suggest the following hypothesis based on the preceding analysis:

**H8a:**  *Privacy concern negatively affects App users’ trust;*

**H8b:**  *Privacy concern negatively affects App users’ authorizing intention.*

### 3.4. Effects of Trust on Intention to Authorize Personal Information

Trust refers to the favorable beliefs that potential trustees have about a particular provider or transaction environment prior to an information interaction, which reduces the uncertainty of the exchange and leads to trusting intentions and trusting behaviors [90]. The important role of trust in information sharing and personal information disclosure has received extensive attention from researchers, and most studies have concluded that trust can positively contribute to users’ willingness to disclose information [41,44,91]. However, because individual behavioral willingness is influenced by the interaction of many factors, some studies showed that the role of trust on individuals’ willingness in the mobile network environment is not significant [92]. In this paper, we define trust as a positive expectation of mobile App users regarding the potential loss of privacy caused by authorizing personal information. When users authorize personal information, they trust the mobile App provider, and, especially when it is difficult to assess whether the App features or services can meet their needs and whether the company can protect personal privacy information well, users’ trust in the App provider will positively contribute to their privacy authorization intention. Accordingly, we propose the following hypothesis:

**H9:**  *Trust positively affects App users’ authorizing intention.*

**Figure 1:**
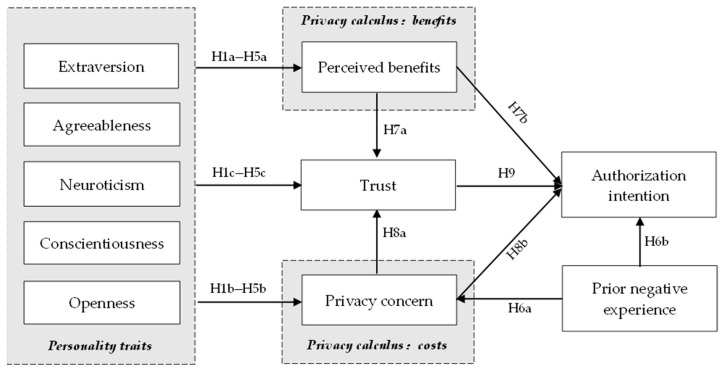
Conceptual model.

Figure 1 depicts the study model for this project, which is based on the assumptions listed above: 

## 4. Methodology

### 4.1. Measurement Development

The research model was validated using a questionnaire, and all variables were obtained from prior studies with modest changes to individual items in our context. Except for one item of conscientiousness, which was assessed in reverse order (i.e., low to high scores from “completely agree” to “fully disagree”), all items were scored on a seven-point Likert scale, while the remaining items were scored from low to high order. The remaining questions were graded on a scale of “totally disagree” to “absolutely agree.” We used Pentina et al.’s [16] existing scale to examine users’ perceived benefits in three dimensions: information source, leisure, and social interaction. At the same time, to keep the questionnaire short, we chose the top three first-order factor load of items to measure perceived benefits. The privacy concern scale was modified from Xu et al. [88]. The simplified scales of the Big Five personality traits are based on Zhou and Lu [45], Junglas et al. [40], and Davis and Yi’s [93] relevant scales. This abbreviated version is more suitable to the analysis of general social attitudes and phenomena than the traditional scale from Goldberg, and its reliability has been frequently proven in the field of privacy research [51]. In the measurement of prior negative experience, we used the measure items in the study of Xu et al. [88] and Li et al. [79]. A pre-test of 30 students who had previously had experiences with App information authorization was performed first. Following their feedback, certain items were amended and improved, and a formal questionnaire was created, as shown in Appendix B. 

### 4.2. Sample and Data Collection Procedure

The official questionnaire was distributed through a unified questionnaire distribution platform. Then, it was circulated utilizing social media platforms such as WeChat friends circle, Sina Weibo, and university BBS. To guarantee that each respondent clearly understood the questionnaire contents, we first introduced the respondents to background information of mobile APP personal information authorization and an explanation of the notion of relevant variables before commencing the questionnaire responses. The survey was split into two sections. The first section of the survey aimed to collect respondents’ personal information, such as gender, age, and educational level, as well as statistical data on the number of applications used and the frequency with which they were authorized. The second part explored the structural links between all of the variables in the study. It was only for those who had ever authorized personal information through a mobile App. Hence, the first question in the questionnaire was “Have you ever permitted or disclosed personal information through a mobile App?” The questionnaire URL link was instantly closed if the response was “No.” We intercepted many authorization pop-ups from “Kugou Music”, a music software that provides music information services, assists users in entertaining themselves, and allows them to exchange music with friends, which may effectively reconstruct the scenario of an App information request, allowing respondents to recall the authorization process and feelings more quickly. In order to increase the effective response rate, we gave each participant a random bonus.

In this paper, we received a total of 533 initial questionnaires, and then we screened out invalid questionnaires in terms of respondents’ response time and options. We regarded them as invalid questionnaires if the respondent took less than one minute to answer or had more than 10 consecutive questions with the same answer, which meant that he/her answered the questions too mechanically or randomly. Finally, we screened 455 valid questionnaires with a return rate of 85.3%. This questionnaire had 37 items, which is in line with the recommendation of Bender and Chou [94] that the total number of factor measures should preferably be kept at 1:10 with the total sample size, indicating that the sample size of this chapter is appropriate. The participant statistics are shown in Table 2.

## 5. Data Analysis

Because the perceived benefits of mobile App users are second-order variables consisting of information source, leisure, and social interaction, and there are many discrepancies in the findings of personality traits on users’ privacy perceptions in the prior literature, the effects of personality traits on users’ perceived benefits, privacy concern, and trust in the context of App authorization need to be further explored; therefore, we use the more prediction-oriented partial least squares structural equation model (PLS-SEM) to validate the relationships among the factors [95].

### 5.1. Common Method Variance

Common method variance (CMV) refers to the contamination of “noise” caused by the same data sources, measurement contexts, and characteristics of the items themselves [96], which is a systematic error. The problem was verified by Harman’s one-factor test, in which the presence of homogeneous variance is determined if the variance explained by the first factor is greater than 50%, conversely indicating the absence of common method bias. The maximum variance explained by a single factor in the model is 20.921%, as shown by the EFA analysis in this chapter. Consequently, the results of both the procedural and statistical treatments confirm the absence of common method bias in our data.

### 5.2. Reliability and Validity

To test scale reliability, we used Cronbach’s alpha coefficient and reliability (CR) as two main indicators. Table 2 shows that all of the Cronbach’s alpha and CR values are greater than 0.7, indicating that the constructs are reliable [97]. Furthermore, Fornell and Larcker [98] presented three criteria for determining the convergent validity of the variables: (1) each item’s standardized factor loadings are larger than 0.7 and achieve a significant level (*p* < 0.05 or *p* < 0.01); (2) all variables’ composite reliability (CR) can surpass 0.7; and (3) each variable’s average extracted variance (AVE) can exceed 0.6. Table 3 shows that all of the constructs’ standardized factor loadings, CR, and AVE values are above the prior studies’ recommended thresholds, showing that the scale is highly reliable and convergent.

Table 4 displays the discriminant validity of constructs. The AVE square root of each variable is greater than the correlation coefficient between this variable and the others, indicating that the research model has adequate discriminant validity.

### 5.3. Second-Order Factor Model

In this study, we define perceived benefits as a reflective second-order variable and evaluate App users’ perceived benefits in three dimensions: information source, leisure, and social interaction. The repeated indicator approach, also known as the “hierarchical component model,” was used to examine the second-order variables [99]. The CR of perceived benefits was 0.907 and the AVE was 0.549, both of which were higher than the essential levels of 0.7 and 0.5 [98]. Furthermore, the path coefficients of perceived benefits to information source, leisure, and social interaction were all greater than 0.7, with decidability coefficients of R^2^ of 0.630, 0.645, and 0.532, respectively, demonstrating that perceived benefits are operationalized as a well-constructed second-order model. Table 5 shows the results.

### 5.4. Data Analysis and Results

The R^2^ value of the coefficient of determination, the cross-validation redundancy Q^2^, and the significance level of the path coefficient are all used to evaluate the PLS-SEM structural equation model [100]. R^2^ is a useful metric for assessing a model’s explanatory power, as it indicates how well endogenous latent variables can be explained. Perceived advantage, privacy concern, trust, and intention to authorize personal information are the endogenous latent variables in the model. The R^2^ value of personal information authorization intention in this paper is 0.335, which is higher than Hair’s [101] estimated explanatory power for the dependent variable in the consumer behavior domain. Perceived benefits, privacy concern, and trust all have R^2^ values above 0.1, showing that the model has some explanatory power for the above endogenous latent variables [102]. As indicated in Table 6, the structural model’s predictive relevance indicator, Q^2^, is greater than 0, indicating that the model has good predictive correlation. Furthermore, the model’s standardized residual root mean square (SRMR) is 0.078, which is less than the 0.08 threshold value and passes the PLS-SEM model fitness condition.

**Figure 2:**
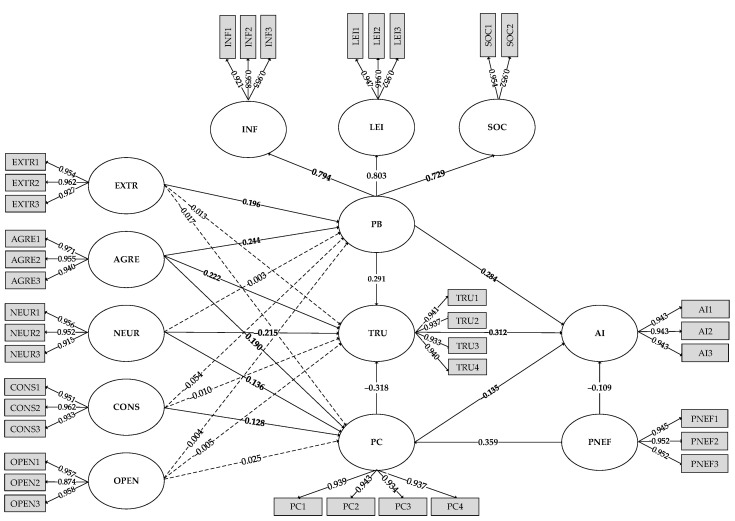
Path analysis coefficient.

The link between the path coefficients of the variables is depicted in Figure 2, and the findings of the 22 hypotheses are analyzed in the Table 7.

(1) The extroversion of users’ personalities influences their perceived App benefits, with a standardized path coefficient of 0.196 for both and a significant path of influence (*t* = 4.378, *p* = 0.000 < 0.01), but extroversion has no effect on users’ privacy concern and trust (*t* = 0.441, *p* = 0.659 > 0.05; *t* = 0.303, *p* = 0.762 > 0.05). Users with agreeable personalities have standardized path coefficients of 0.244, 0.190, and 0.222 for all three paths, with a 0.01 level of significance (*t* = 5.402, *p* = 0.000 < 0.01; *t* = 3.831, *p* = 0.000 < 0.01; *t* = 5.576, *p* = 0.000 < 0.01). The personality trait of neuroticism increases privacy concern (*t* = 3.042, *p* = 0.002 < 0.01) and lessens trust (*t* = 5.012, *p* = 0.000 < 0.01), with path coefficients of 0.136 and −0.215, respectively, but has no effect on perceived benefits (*t* = 0.069, *p* = 0.945 > 0.05). Conscientiousness has no influence on perceived benefits or trust (*t* = 1.128, *p* = 0.260 > 0.05; *t* = 0.260, *p* = 0.795 > 0.05), but it does have a positive effect on privacy concern (*t* = 3.112, *p* = 0.002 < 0.01), with a path coefficient of 0.128. Benefits, privacy, and trust are not affected among users with an open personality (*t* = 0.085, *p* = 0.932 > 0.05; *t* = 0.551, *p* = 0.582 > 0.05; *t* = 0.105, *p* = 0.917 > 0.05).
(2) The standardized path coefficient of prior negative experience on users’ privacy concern is 0.359, with a 0.01 level of significance (*t* = 7.856, *p* = 0.000 < 0.01), indicating that prior negative experience could have a significant positive influence on users’ privacy concern. Correspondingly, the standardized path coefficient of prior negative experience on users’ intention to authorize is −0.109, with a 0.01 level of significance (*t* = 2.746, *p* = 0.000 < 0.01), demonstrating that there is a negative relationship between past bad experience and App users’ willingness to authorize their personal information.
(3) The standardized path coefficient values for users’ perceived benefits on trust and willingness to authorize are 0.291 and 0.284, respectively, and both paths show significance at the 0.01 level (*t* = 7.546, *p* = 0.000 < 0.01; *t* = 7.198, *p* = 0.000 < 0.01), demonstrating that users’ perceived benefits have a significant positive impact. Furthermore, the standardized path coefficient values of users’ privacy concern on their trust and willingness to authorize information are −0.318 and −0.135, respectively, with 0.01 level of significance (*t* = 8.347, *p* = 0.000 < 0.01; *t* = 3.031, *p* = 0.002 < 0.01), indicating that privacy concern has a significant negative impact on both users’ trust and their authorizing intention. Finally, the standardized path coefficient value of user trust on their desire to authorize is 0.312, with a significance level of 0.01 (*t* = 6.816, *p* = 0.000 < 0.01), demonstrating that user trust can have a significant positive influence relationship on users’ willingness to authorize information.

## 6. Discussion and Implications

### 6.1. Discussion

This study looks into the impact of five primary personality qualities on users’ privacy calculations and trust, as well as the various elements that influence their willingness to authorize. It shows that the positive effects of user trust (H9) and perceived benefits (H7b) are more significant than the constraining effect of privacy concern on App users’ willingness to authorize personal information (H8b), and these findings are proved in studies by Xu et al. [103], Zlatolas et al. [104], and Wakefield [82], which demonstrated that users’ functional expectations of a product, as well as their trust in it, are more likely to prompt them to make quick decisions.

Except for extraversion and agreeableness, which have a significant positive effect on users’ perceived benefits (H1a and H2a), none of the other five personality traits have a significant effect on users’ perceived benefits (H1a, H2a, H4a, H5a), which is consistent with Pentina et al.’s [16] findings on the five personalities and perceived benefits. Users with the traits of agreeableness, neuroticism, and conscientiousness, on the other hand, are more concerned about privacy risks (H1b, H2b, H3b), findings that were confirmed in Bansal et al. [44] and Junglas et al.’s [40] studies, but the relationship between extraversion, openness, and privacy concern is not significant (H4b, H5b). Perhaps this is because, according to Junglas et al. [40], extroversion focuses more on an individual’s social tendencies, particularly in their interactions with others, whereas the privacy authorization process for Apps is primarily a permission mechanism that does not involve interactions with others, so App users’ traits associated with extroversion may not be fully reflected in this scenario and thus have little impact on their privacy concern. In addition, Costa and McCrae [39] have expressed that openness is a double-edged sword. People with openness traits may hold both higher positive perceptions and negative emotions, and their feelings about privacy authorization are not focused on a single benefit, cost, or trust dimension. Hence, the effect of openness on all three may be insignificant.

### 6.2. Implications for Theory

First, this study gives researchers a new viewpoint on App users’ privacy behaviors. While previous research has frequently regarded privacy concern as the most representative privacy proxy factor, the empirical findings in this study reveal that focusing just on users’ privacy concern does not effectively predict their privacy intention. The outcomes of this study support the privacy calculus theory, demonstrating that privacy benefits and cost have mutual checks and balances on App users’ willingness to authorize their information, and that trust has a positive impact on their willingness as well. Furthermore, by empirical analysis, this paper addresses the “privacy paradox” phenomena and its underlying motivation, i.e., users may disregard privacy concern and prefer to disclose personal information in order to meet certain functional requirements.

Meanwhile, we evaluated the effects of various users’ personality traits on their privacy perceptions, and we argued for the mediating function of privacy concern by considering individuals’ prior negative experiences as crucial individual determinants. Although the results of previous studies on the effect of the Big Five personality traits and privacy perception characteristics have varied, related study has become one of the most important areas of privacy research in recent years. This paper examines the effects of agreeableness, neuroticism, conscientiousness, extraversion, and openness on one’s perceived benefits, privacy concern, and trust in the context of App privacy empowerment, allowing future researchers to gain a better understanding of the antecedents of individual privacy feelings and enriching research ideas and research.

### 6.3. Implications for Providers

This research has significant implications for App providers’ policy formulation and privacy management procedures.

To begin with, the functional services that applications can provide have a significant impact on user information permission decisions and can even persuade users to disregard their privacy concern, resulting in the “privacy paradox” phenomenon of separating privacy attitudes and decision-making behaviors. As a result, App developers should boost users’ willingness to contribute personal information and improve their perceptions of benefits in all areas. First, App developers should focus on improving the App’s fundamental functions, such as providing users with timely and accurate information or its practical utility. Second, as people’s living standards rise and their need for entertainment and leisure increases, App developers must review their Apps’ entertainment features, improve user immersion and pleasure, and provide in-depth and connotative entertainment content. Finally, it is a new thread of thought to improve Apps’ information-sharing capabilities and make it easier for more people to communicate with one another.

Furthermore, App providers should respect users’ privacy rights and interests, minimize privacy infringement to the greatest extent possible, mitigate users’ privacy concerns, and work to increase users’ trust in mobile Apps. On the one hand, precise and detailed privacy policies that are clear and easy to understand can be developed, and technical capabilities such as virtual identity authorization, account vulnerability scanning, and illegal information interception can be enhanced to reduce the insecurity of users’ information authorization. On the other hand, reducing the number of permission requests for highly sensitive information is essential. When collecting highly sensitive information, App providers should pay special attention to stating the purpose of access and the information processing process in the privacy policy in order to lower users’ risk perception levels. In addition, App developers should follow the “least necessary” approach to limit the collection of user information and reduce the extent of data collected and provide individual authorization pop-ups when specific permissions are required.

Last but not least, users with different personalities have different functional expectations, privacy concern, and trust, so App providers should effectively combine user characteristics, fully consider their differentiated needs, establish a classification management mechanism for privacy services, and give App users with different personality traits the option of “function first” or “privacy first.” For example, highly neurotic App users are likely to be overly concerned about privacy issues while also distrustful of personal information authorization. App providers can provide more privacy setting options for them to choose from as well as extend their authorization options without forcing them to authorize all at once. Despite the fact that users with highly agreeable characteristics are more inclined to accept information permission and expect a functional experience, they are concerned about privacy issues. As a result, the App provider’s sincere presentation and notice, such as delivering a privacy risk warning at the time of information authorization or outlining the enterprise’s countermeasures to protect users’ personal information, can have a beneficial influence on their privacy decisions.

## 7. Limitation and Future Direction

When compared to studies examining the effect between constructs supported by a large amount of literature and fixed conclusions, the number of studies on the influence of personality traits and privacy calculus, as well as the relationship between personality traits and trust, is currently small, which may result in insufficient references and affect the study’s rigor. However, this is a good model for future research because there is still a lot of room for expanding and improving research on personality traits and users’ privacy perceptions and privacy decisions.

## Figures and Tables

**Table 1:** Research related to personality traits and privacy-perception factors.

Authors	Context	Personality Traits	Investigated Constructs		
			Perceived Benefits	Privacy Concern	Trust
Pentina et al. [29]	Mobile apps	Big Five Factors	√	√	
Yeh et al. [46]	E-commerce	Big Five Factors		√	
Zhou and Lu [45]	Mobile Commerce	Big Five Factors	√		√
Agyei et al. [47]	Mobile Banking	Big Five Factors	√		
Bansal et al. [44]	Online Finance/Health/E-commerce	Big Five Factors		√	√
Koohikamali et al. [48]	Social Network Sites (SNS)	Agreeableness Extraversion		√	
Mouakket and Sun [49]	Social Network Sites (SNS)	Big Five Factors	√		
Schyff et al. [50]	Facebook	Big Five Factors		√	
Deng et al. [41]	Social Media	Agreeableness Conscientiousness			√
Pour and Taheri [51]	Knowledge Sharing	Big Five Factors			√
Mooradian et al. [52]	Knowledge Sharing	Agreeableness			√
Junglas et al. [40]	Location-based Services	Big Five Factors		√	
Bawack et al. [53]	Voice shopping	Big Five Factors		√	√

“√” denotes that personality traits were used as predictors of this privacy-perception constructs.

**Table 2:** Respondent personal information.

Characteristics	Items	Frequency	Percentage
Gender	Male	237	52.1%
	Female	218	47.9%
Age (years)	<20	108	23.8%
	20~30	169	37.1%
	30~45	103	22.6%
	>45	75	16.5%
Monthly Profit (RMB)	Less than 3000	152	33.4%
	3000~4999	102	22.4%
	5000~7999	114	25.1%
	More than 8000	87	19.1%
Education	Less than high school	33	7.3%
	College or university	308	67.6%
	Advanced degree	114	25.1%
Operating System	Android	325	63.7%
	iPhone OS	185	36.3%
Frequency of authorization (times a week)	Less than 5	103	22.6%
	5~10	152	33.4%
	11~20	123	27%
	More than 20	77	17%

**Table 3:** Standardized item loadings, AVE, CR and Alpha values.

Factor		Item	Standardized Item Loading	CR	Cronbach’s α	AVE
Extraversion		EXTR1	0.953	0.963	0.943	0.898
		EXTR2	0.962			
		EXTR3	0.927			
Agreeableness		AGRE1	0.971	0.969	0.952	0.913
		AGRE2	0.955			
		AGRE3	0.940			
Neuroticism		NEUR1	0.956	0.959	0.936	0.886
		NEUR2	0.952			
		NEUR3	0.915			
Conscientiousness		CONS1	0.951	0.964	0.945	0.900
		CONS2	0.962			
		CONS3	0.933			
Openness		OPEN1	0.957	0.951	0.952	0.866
		OPEN2	0.874			
		OPEN3	0.958			
Perceived Benefits	Information source	INF1	0.921	0.961	0.893	0.940
		INF2	0.958			
		INF3	0.955			
	Leisure	LEI1	0.947	0.964	0.900	0.944
		LEI2	0.946			
		LEI3	0.952			
	Social interaction	SOC1	0.954	0.952	0.909	0.900
		SOC2	0.952			
Privacy Concern		PC1	0.939	0.967	0.955	0.880
		PC2	0.943			
		PC3	0.934			
		PC4	0.937			
Trust		TRU1	0.941	0.967	0.954	0.880
		TRU2	0.937			
		TRU3	0.933			
		TRU4	0.940			
Intention to authorize		AI1	0.943	0.960	0.938	0.889
		AI2	0.943			
		AI3	0.943			
Prior negative experience		PPIE1	0.945	0.965	0.945	0.902
		PPIE2	0.952			
		PPIE3	0.952			

**Table 4:** Correlation coefficients and square root of AVE.

	EXTR	AGRE	NEUR	CONS	OPEN	INF	LEI	SOC	PC	TRU	AI	PNEF
EXTR	***0.947***											
AGRE	0.110	***0.956***										
NEUR	−0.108	0.043	***0.941***									
CONS	0.079	−0.040	−0.090	***0.949***								
OPEN	−0.094	−0.058	0.024	−0.012	***0.931***							
INF	0.167	0.218	0.012	0.020	−0.001	***0.945***						
LEI	0.176	0.182	0.012	−0.057	−0.079	0.380	***0.949***					
SOC	0.165	0.235	−0.046	−0.095	0.034	0.405	0.444	***0.953***				
PC	−0.019	0.215	0.165	0.142	−0.016	−0.075	−0.008	−0.046	***0.938***			
TRU	0.103	0.222	−0.256	−0.060	−0.024	0.335	0.247	0.270	−0.322	***0.938***		
AI	0.157	0.097	−0.081	−0.005	−0.045	0.366	0.291	0.309	−0.294	0.482	***0.943***	
PNEF	−0.045	0.078	0.084	0.099	−0.091	−0.086	−0.053	−0.068	0.396	−0.209	−0.253	***0.949***

Note: the bold italic diagonal numbers are the square root of AVE.

**Table 5:** Assessment of the higher-order factor model.

Second-Order Factor	First-Order Factor	CR	AVE	Path Coefficient	R^2^
Perceived Benefits	Information Source	0.907	0.549	0.794 *** (*t* = 36.503)	0.630
	Leisure			0.803 *** (*t* = 45.550)	0.645
	Social Interaction			0.729 *** (*t* = 30.806)	0.532

Note: *** *p* < 0.001.

**Table 6:** Results of Q^2^ values and R^2^ values.

Factor	SSO	SSE	Q^2^ (=1 − SSE/SSO)	R^2^
Perceived Benefits	3640.000	3429.237	0.058	0.111
Privacy Concern	1820.000	478.515	0.188	0.223
Trust	1820.000	1327.706	0.270	0.312
Intention to Authorize	1365.000	965.616	0.293	0.335

**Table 7:** Summary of hypotheses testing results.

Hypotheses	*t*-Value	Standard Deviation	*p*-Value	Path Coefficients	Results
H1a	4.378	0.045	0.000	0.196	Supported
H1b	0.441	0.038	0.659	−0.017	Unsupported
H1c	0.303	0.043	0.762	−0.013	Unsupported
H2a	5.402	0.045	0.000	0.244	Supported
H2b	3.831	0.049	0.000	0.190	Supported
H2c	5.576	0.040	0.000	0.222	Supported
H3a	0.069	0.041	0.945	0.003	Unsupported
H3b	3.042	0.045	0.002	0.136	Supported
H3c	5.012	0.043	0.000	−0.215	Supported
H4a	1.128	0.048	0.260	−0.054	Unsupported
H4b	3.112	0.041	0.002	0.128	Supported
H4c	0.260	0.040	0.795	−0.010	Unsupported
H5a	0.085	0.052	0.932	0.004	Unsupported
H5b	0.551	0.044	0.582	0.025	Unsupported
H5c	0.105	0.044	0.917	−0.005	Unsupported
H6a	7.856	0.046	0.000	0.359	Supported
H6b	2.746	0.040	0.006	−0.109	Supported
H7a	7.546	0.039	0.000	0.291	Supported
H7b	7.198	0.039	0.000	0.284	Supported
H8a	8.347	0.038	0.000	−0.318	Supported
H8b	3.031	0.044	0.003	−0.135	Supported
H9	6.816	0.046	0.000	0.312	Supported

## Data Availability

Not applicable.

## Appendix A

**Table A1:** Positivist IS Studies on Privacy Calculus.

Authors and Years	Context	Privacy Calculus Related Constructs			Major Findings
		Benefit	Cost	Outcome	
Dinev and Hart (2006) [6]	E-commerce	Personal Internet interest	Internet privacy concern	Willingness to provide personal information to transact on the Internet	This research attempts to better understand the cumulative influence of Internet trust and personal Internet interest are important to outweigh privacy concern in the decision to disclose personal information through online transactions.
Yeh et al. (2018) [46]	E-commerce	Extrinsic rewards	Information privacy concern	Willingness to provide personal information	Information privacy concern did not significantly affect users’ willingness to provide personal information in the privacy calculation mechanism; however, extrinsic rewards directly affected users’ disclosure intention.
Xu et al. (2011) [105]	location-aware marketing (LAM)	Perceived benefits of information disclosure	Perceived risks of information disclosure	Perceived value of information disclosure	The positive relationship between perceived benefits and perceived value, and the negative relationship between privacy risk and perceived value were found significant in both covert and overt approaches.
Gutierrez et al. (2019) [23]	Mobile location-based advertising (MLBA)	PersonalizationMonetary rewards	Internet privacy Concern Intrusiveness	Acceptance of MLBA	While internet privacy concerns is a primary determinant of acceptance intentions towards MLBA, but monetary rewards and intrusiveness have a notably stronger impact on it.
Jiang et al. (2013) [24]	Online social interaction	Social rewards	Privacy concern	Self-disclosure Misrepresentation	Drawing on the privacy calculus perspective, the interesting roles of privacy concerns and social rewards in synchronous online social interactions are developed and validated.
Zlatolas et al. (2015) [104]	Social Network Sites (SNS)	Privacy value	Privacy concern	Disclosure intention	There is a significant relationship between privacy value/privacy concerns and self-disclosure, and the privacy value is more influential than privacy concern in determining users’ self-disclosure.
Min and Kim (2015) [84]	Social Network Sites (SNS)	Behavior enticements *(identification)* *(internalization)* *(compliance)*	Privacy concern	Intentions to give personal information Continuous intentions to use SNS	The findings show that privacy concerns severely inhibit people from providing information in SNS, and, besides the subjective social norms, the other two behavior enticements have been proved to promote the disclosure intention and behavior.
Ma et al. (2021) [106]	Social Network Sites (SNS)	Perceived usefulness Perceived controllability	Perceived severity Perceived intrusion	Self-disclosure intentions	The findings confirmed that individuals’ perceptions of severity and intrusion influenced users’ self-disclosure intentions, and predicting benefit constructs such as perceived usefulness and perceived controllability were found to positively influence self-disclosure intentions.
Sun (2021) [107]	Social Network Sites (SNS)	Self-expression Life documentation Social rewards	Privacy risks	Intention to disclose	The findings suggest that when users believe that disclosing personal information will meet their needs for social rewards, self-expression, or life documentation, and the privacy risks are low, they will do so.
Pentina et al. (2016) [29]	Mobile apps	Perceived benefits	Perceived privacy concern	Mobile apps’ use intention Mobile apps’ use	The perceived benefits are partially identified as drivers of a wide range of mobile app adoption and use in both US and China, but the effect of privacy concerns on the adoption is not obvious.
Wang et al. (2016) [85]	Mobile apps	Perceived benefits	Perceived risks	Intention to disclose via Mobile application	Drawing on the privacy calculus theory, this research proved that the lure of perceived benefits is greater than the loss of perceived costs when users are weighing up whether to disclose their information or not.
Cho et al. (2018) [108]	Wearable device & service	Perceived value	Perceived privacy concern	Self-disclosure intention	The perceived value had a greater impact than perceived privacy concern on information disclosure, and the perceived privacy concern decreased the perceived value from a wearable device user’s perspective.
Widjaja et al. (2019) [21]	Cloud storage	Personal interest Perceived usefulness	Privacy concern Privacy risk Security concerns	Willingness to put personal information Trust	Cloud storage users’ willingness to put personal information is highly influenced by trust, perceived costs, and perceived benefits.
Bui Thanh Khoa (2020) [109]	Mobile banking services	Perceived credibility Information interest Perceived control	Privacy concern Perceived vulnerability	Perceived value	It was found that all the constructs of perceived benefits and perceived costs have a remarkable effect on perceived value in the mobile banking services context.
Duan and Deng, H. (2021) [110]	Contact tracing apps	Performance expectancy	Perceived privacy risk	Perceived value of information disclosure	The analysis result confirmed that performance expectancy and perceived privacy risks are indirectly significant on the adoption through the influence of perceived value of information disclosure.
Zhu et al. (2021) [111]	mHealth apps	Perceived benefits	Privacy concern	Disclosure intention	When determining information disclosure, the users’ benefits perception for using mHealth applications is two or three times more influential than their privacy concerns.
Zhang et al. (2018) [9]	Online health communities	Perceived informational support Perceived emotional support	Privacy concern	Disclosure intention	Results indicate that health information privacy concerns, together with informational and emotional support, significantly influence personal health information (PHI) disclosure intention.

## Appendix B

**Table A2:** The final items and sources of each construct.

Factor		Item	Wording
Extraversion		EXTR1	I like to be surrounded by friends
		EXTR2	I am always happy and energetic
		EXTR3	I am passionate about others
Agreeableness		AGRE1	I have a tolerant nature
		AGRE2	I am courteous and friendly to others
		AGRE3	I like to work with others
Neuroticism		NEUR1	I am easily anxious
		NEUR2	My emotional ups and downs are numerous.
		NEUR3	I am constantly worried that something bad will occur.
Conscientiousness		CONS1	I am good at developing plans and carrying them out.
		CONS2	I am meticulous when it comes to completing tasks
		CONS3	I consider myself disorganized and irresponsible (R)
Openness		OPEN1	I am curious about new and exciting things
		OPEN2	I like to come up with new ideas and new thoughts
		OPEN3	I like to break the rules and experience new things
Perceived benefits	Information source	INF1	I get information more easily through the mobile app
		INF2	I get better products and services through the mobile app
		INF3	Mobile apps provide me with the latest information and news
	Leisure	LEI1	I can relax more by using mobile apps
		LEI2	Mobile apps can make my daily life more leisurely
		LEI3	Mobile apps enable me to get more entertainment
	Social interaction	SOC1	I can interact with others through the use of mobile apps
		SOC2	I can stay connected to the community by using mobile apps
Privacy Concern		PC1	I am concerned that this app will over-collect my personal information
		PC2	I am concerned that the personal information stored in this app could be misused
		PC3	I am concerned that this app will leak my personal information to unauthorized third-party agencies
		PC4	I am concerned that my personal information is at risk due to errors and omissions of data users
Trust		TRU1	This app is trustworthy in authorizing my personal information.
		TRU2	I trust that this app will tell the truth and fulfill promises related to my personal information
		TRU3	I trust that this app will keep my best interests in mind when dealing with personal information
		TRU4	I trust that this app is always honest with users when it comes to using the information that I would provide
Intention to authorize		AI1	At the right time, I intend to authorize my personal information to the apps’ background
		AI2	In the future, I will probably authorize my personal information to the apps’ background
		AI3	In the future, I would like to authorize my personal information to the apps’ background
Prior negative experience		PPIE1	While utilizing existing mobile apps, I have been the victim of numerous privacy intrusions
		PPIE2	Apps have regularly collected enormous amounts of personal information from me
		PPIE3	Apps have regularly used my personal information without my permission
